# Bridging the Gap: Addressing Barriers in Bariatric Surgery Training in Pakistan

**DOI:** 10.7759/cureus.87613

**Published:** 2025-07-09

**Authors:** Haseeb Javed Khan, Abdul Kamil Ghumman, Tahir Yunus

**Affiliations:** 1 Department of Surgery, Evercare Hospital, Lahore, PAK; 2 Hepatopancreatobiliary and Liver Transplant Surgery, Sheikh Zayed Hospital Lahore, Lahore, PAK

**Keywords:** bariatric surgery, health policy, medical education, obesity, surgery in pakistan

## Abstract

Background

Obesity is a growing public health crisis in Pakistan, with increasing numbers of individuals experiencing obesity related complications such as diabetes and cardiovascular disease. Metabolic and bariatric surgery (MBS) is a well-established, evidence-based intervention for managing severe obesity. However, its utilization remains suboptimal in many low- and middle-income countries, including Pakistan. Despite an increasing clinical need, MBS training and practice remain underdeveloped in Pakistan. This study aims to identify key barriers impeding MBS development and explore potential solutions through a nationwide survey of surgical professionals.

Methods

A survey was distributed among surgery residents, fellows, and consultants in major hospitals across Pakistan. Designed using Google Forms, the questionnaire included consent and biodata forms, along with eight questions each addressing barriers and solutions in MBS training and practice. Responses were evaluated using a 5-point Likert scale. Reliability analysis, descriptive statistics, and the Pearson chi-square test were utilized for data analysis.

Results

We received 55 responses from 25 hospitals nationwide between February and March 2024. Participants, with an average age of 37.7 ± 10.2 years and 21.8% female representation, included residents (38.2%), fellows (20%), and consultant surgeons (41.8%). Notably, 27.3% had formal MBS training, while 12.7% had over five years of experience. Key barriers identified encompassed limited public and surgeon awareness, financial limitations, unclear guidelines, faculty shortages, societal stigma, and policy gaps regarding MBS as a treatment for obesity. Proposed solutions included media campaigns, international collaborations, exchange programs, policy reforms, establishing a national MBS registry and center of excellence, mentorship programs, and tailored guidelines by the Pakistan Society of Metabolic and Bariatric Surgery (PSMBS).

Conclusion

There is an urgent need to address the multifactorial barriers hindering MBS expansion in Pakistan. Strengthening training infrastructure, enhancing public and professional awareness, and developing a robust policy framework are critical steps toward improving access to surgical care for obesity and reducing the national burden of related comorbidities.

## Introduction

Pakistan, with an estimated population of 240 million in 2023, is facing a growing public health crisis driven by obesity and diabetes [1]. The World Health Organization data shows that 58.1% of Pakistanis are overweight, with 43.9% classified as obese, whereas the prevalence of diabetes has reached 26.7% which is among the highest globally [2]. These rising trends are attributed to rapid urbanization, sedentary lifestyles, and changes in dietary habits, reflecting a broader epidemiological transition toward non-communicable diseases (NCDs).

These alarming statistics have created an urgent need for effective interventions targeting obesity and its complications. In this context, metabolic and bariatric surgery (MBS) emerges as a crucial but underutilized tool in Pakistan’s health system. MBS is globally recognized as the most effective intervention for morbid obesity and related metabolic conditions, such as type 2 diabetes, hypertension, and dyslipidemia. MBS not only induces durable weight loss but also improves quality of life and reduces long-term mortality [3-5]. However, despite compelling evidence and the clear demand created by Pakistan’s metabolic disease burden, the development and integration of bariatric surgery into the national health system remain limited.

As of 2023, Pakistan has fewer than 100 trained bariatric surgeons and only two accredited fellowship programs. These limitations have resulted in inadequate access to care, delayed referrals, limited institutional growth, and poor surgical outcomes. In contrast, many high-income countries have national MBS registries, structured training pathways, and reimbursement models that ensure equitable and evidence-based delivery of care.

Given the mounting burden of obesity-related diseases and the underutilization of an effective therapeutic option, it is imperative to understand the specific barriers limiting MBS training and practice in Pakistan. Mitigating these barriers is critical not only for expanding access to life-altering surgical care but also for reducing the long-term burden of non-communicable diseases on the healthcare system. Enhancing MBS capacity can lead to earlier interventions, fewer complications from advanced diabetes and cardiovascular disease, and more cost-effective management of obesity at the population level. This study aims to identify these obstacles and suggest feasible, context-sensitive solutions that could advance surgical care for obesity and metabolic diseases in low- and middle-income countries (LMICs) such as Pakistan.

The abstract of this paper was presented at the Association of Surgeons in Training (ASIT) annual conference on March 7, 2025.

## Materials and methods

Study design and setting

This cross-sectional, questionnaire-based study was conducted between February and March 2024. It aimed to assess barriers to MBS training and practice in Pakistan. Data were collected from surgical professionals affiliated with 25 major public and private tertiary care hospitals across the country, ensuring a broad and representative sample of the national surgical landscape.

Participant recruitment

Eligible participants included surgical residents enrolled in accredited general surgery training programs, general surgery fellows, bariatric surgery fellows, and consultant surgeons practicing in Pakistan with an interest in general, gastrointestinal, or bariatric surgery. Participants were required to voluntarily consent to the study and complete the online survey. Individuals were excluded if they were medical students, house officers, or interns not currently in surgical training; surgeons practicing outside Pakistan; or if they submitted incomplete or duplicate responses. Non-clinical personnel or individuals without a verifiable surgical background were also excluded to ensure the relevance and reliability of the data. Recruitment was conducted via institutional contacts, surgical societies, and professional platforms such as WhatsApp and email. The convenience sampling was used for participant selection.

Sample size calculation

The sample size was calculated using G*Power software (version 3.1.9.7) for chi-square analysis with an expected moderate effect size (w = 0.3), power of 80%, and alpha of 0.05. For a 5-point Likert response (df = 4), the minimum required sample size was 50. Our study achieved a final sample of 55 participants, fulfilling this requirement for adequate statistical power.

Survey instrument

**Figure 1:**
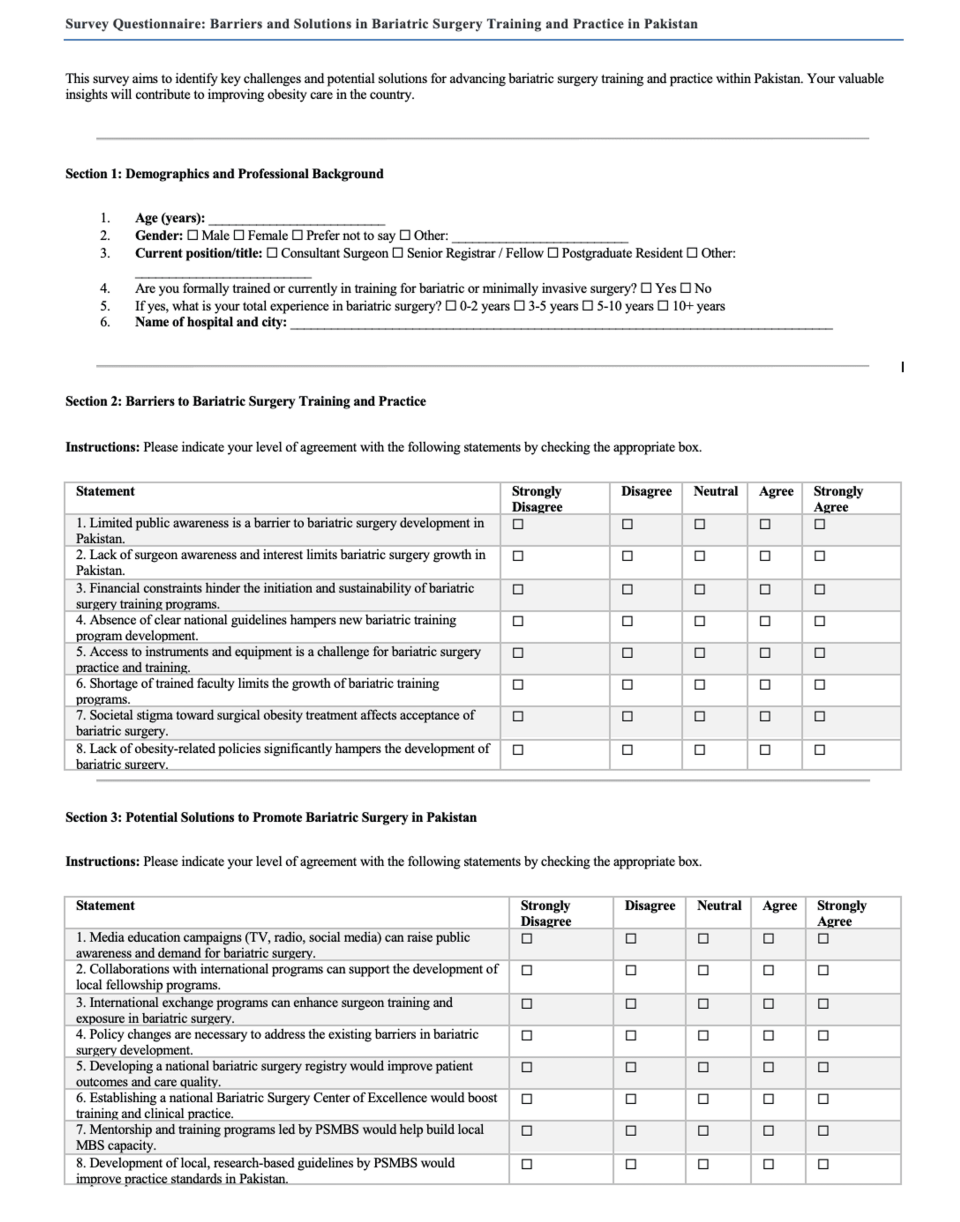
Questionnaire of Barriers and Solutions in Bariatric Surgery Training and Practice in Pakistan

Data were collected using a structured, self-administered questionnaire developed in Google Forms (Alphabet Inc., Mountain View, CA, USA). The survey included four main sections: informed consent, participant demographics and professional background, assessment of perceived barriers to MBS training and practice and evaluation of potential solutions. Eight barrier items and eight solution items were included, each rated using a five-point Likert scale (ranging from “strongly disagree” to “strongly agree”) as shown in Figure 1. The questionnaire was developed based on a review of relevant literature and expert consultation. It was peer reviewed by five surgical professionals for clarity, content validity, and relevance. Minor modifications were made accordingly. The questionnaires were pilot tested for internal validity with Cronbach’s alpha values of 0.7 and 0.8 for barriers and solutions questionnaires respectively.

Ethical considerations

The study was approved by the Institutional Review Committee of Evercare Hospital Lahore under reference number IRC/24/08/002. Informed consent was obtained electronically from all participants before initiating the survey. Confidentiality and anonymity were maintained throughout the data collection and analysis phases. 

Data management and statistical analysis

Survey responses were downloaded from Google Forms and processed using Microsoft Excel (Redmond, WA, USA) and IBM SPSS Statistics version 29 (Armonk, NY, USA). Descriptive statistics were used to summarize participant characteristics and overall response trends. Categorical variables were analyzed using chi-square (χ²) tests to examine associations between participant characteristics (e.g., training level, institutional affiliation) and their responses to barrier and solution items. The chi-square test was chosen due to its appropriateness for analyzing relationships between categorical variables. A two-tailed p-value of less than 0.05 was considered statistically significant. Normality testing was conducted for continuous variables using the Shapiro-Wilk test. 

## Results

Participant characteristics

A total of 55 responses were received. Participants included 38.2% surgical residents, 20% fellows, and 41.8% consultants. The average age was 37.7 ± 10.2 years, and 21.8% were female. Only 27.3% of respondents had formal training in MBS, while just 12.7% reported more than five years of experience in the field. These figures underscore the limited exposure and specialization in bariatric surgery across Pakistan’s surgical community.

Identified barriers to MBS development

Participants were asked to evaluate several barriers to MBS practice and training using a 5-point Likert scale. Table 1 summarizes the most common responses and their statistical significance.

**Table 1:** Summary of Perceived Barriers to Metabolic and Bariatric Surgery in Pakistan (n = 55)

Question number	Barrier	Most Common Response	Chi-Square (χ², df)	p-value	Significance
1	Limited public awareness	Agree (29/55)	32.20 (df = 3)	<0.001	Significant
2	Lack of surgeons’ awareness	Agree (21/55)	14.00 (df = 4)	0.007	Significant
3	Financial limitations	Strongly Agree (30/55)	43.26 (df = 3)	<0.001	Significant
4	Unclear guidelines	Agree (26/55)	19.26 (df = 3)	<0.001	Significant
5	Difficulty accessing instruments	Agree (20/55)	4.71 (df = 3)	0.194	Not Significant
6	Shortage of trained faculty	Strongly Agree (28/55)	38.46 (df = 3)	<0.001	Significant
7	Societal stigma	Neutral (16/55)	16.91 (df = 4)	0.002	Significant
8	Lack of policy support	Agree (28/55)	31.91 (df = 3)	<0.001	Significant

Several barriers were found to be statistically significant, including limited public awareness (p < 0.001), lack of surgeon awareness and interest (p = 0.007), financial constraints (p < 0.001), lack of clear guidelines (p < 0.001), faculty shortages (p < 0.001), societal stigma (p = 0.002), and lack of policy support (p < 0.001). Only one item - difficulty accessing instruments - was not statistically significant (p = 0.194), possibly reflecting variability in institutional resources.

These findings highlight multiple systemic gaps within Pakistan’s healthcare infrastructure. The significance of public and professional awareness suggests that obesity and surgical treatment options remain poorly understood. Financial limitations and lack of clear national guidelines point to insufficient governmental and institutional prioritization of obesity treatment. The shortage of trained faculty reflects a broader educational capacity deficit, while stigma and policy inattention signal deep-rooted cultural and regulatory neglect of obesity as a chronic, treatable condition.

Proposed solutions to advance MBS in Pakistan

Respondents were also asked to evaluate proposed interventions for improving bariatric surgery access and training. All suggested solutions showed statistically significant support (p < 0.001), as summarized in Table 2.

**Table 2:** Summary of Proposed Solutions to Improve Metabolic and Bariatric Surgery in Pakistan (n = 55) PSMBS: Pakistan Society of Metabolic and Bariatric Surgery

Q#	Proposed Solution	Most Common Response	Chi-Square (χ², df)	p-value	Significance
1	Media campaigns	Agree (28/55)	38.46 (df = 3)	<0.001	Significant
2	International collaboration	Strongly Agree (29/55)	18.15 (df = 2)	<0.001	Significant
3	International exchange programs	Strongly Agree (33/55)	50.82 (df = 3)	<0.001	Significant
4	Policy changes	Agree (30/55)	15.75 (df = 2)	<0.001	Significant
5	National bariatric surgery registry	Agree (26/55)	16.84 (df = 2)	<0.001	Significant
6	Bariatric surgery centers of excellence	Strongly Agree (30/55)	20.98 (df = 2)	<0.001	Significant
7	Mentorship programs by PSMBS	Agree (25/55)	34.24 (df = 3)	<0.001	Significant
8	Guidelines by PSMBS	Agree (29/55)	36.71 (df = 3)	<0.001	Significant

Most participants strongly agreed that international exchange programs (χ² = 50.82, p < 0.001) and collaboration with international centers (χ² = 18.15, p < 0.001) would enhance training. Media campaigns to raise public awareness (χ² = 38.46, p < 0.001), policy reform (χ² = 15.75, p < 0.001), and establishing a national registry (χ² = 16.84, p < 0.001) were also endorsed as effective strategies. Notably, mentorship initiatives and the development of locally driven clinical guidelines by the Pakistan Society of Metabolic and Bariatric Surgery (PSMBS) received strong support (χ² = 34.24 and 36.71, respectively, p < 0.001).

These preferences suggest a clear demand for structural reform at multiple levels: health policy, surgical training infrastructure, public engagement, and institutional leadership. The strong endorsement of international exchange and registry systems highlights a desire to align Pakistan’s bariatric surgery practices with global standards, while the support for PSMBS-led mentorship and guideline development reflects confidence in local professional leadership.

## Discussion

This study identifies critical structural and perceptual barriers to the development of MBS in Pakistan, while also highlighting potential solutions supported by statistically significant findings. Most participants including surgical residents, fellows, and consultants reported systemic and knowledge-based obstacles that impair the expansion of MBS services nationwide.

Public and professional awareness deficits

Notably, limited public awareness was the most commonly agreed-upon barrier, with 52.7% of participants agreeing that the general population remains poorly informed about bariatric procedures. This is consistent with global studies that indicate a widespread misunderstanding of obesity as a lifestyle choice rather than a chronic disease, especially LMICs [6]. In Pakistan, these gaps in understanding are mirrored within the medical community as well. In our study, 38% of respondents cited poor awareness among healthcare providers as a key issue, supported by regional studies such as a cross-sectional survey from Khyber Pakhtunkhwa, where nearly one in four physicians lacked knowledge of bariatric options and rarely referred eligible patients for surgery [7]. 

Financial and institutional barriers

Financial barriers also showed the highest level of consensus among participants, with a strong majority agreeing that cost is a prohibitive factor for patients. Given that Pakistan lacks public healthcare coverage for surgical management of obesity, bariatric procedures remain out of reach for most patients. Studies from other LMICs confirm that financial access is a key determinant of MBS uptake, especially when surgery is not subsidized or included in public healthcare benefit packages [8-10].

Another statistically significant barrier identified was the shortage of trained bariatric faculty, with 51% of respondents strongly agreeing. This is a major concern given that surgical outcomes in bariatric procedures are closely linked to surgeon experience and institutional volume [11-13]. In Pakistan, there are currently limited structured fellowship programs, and fewer than a dozen institutions perform these surgeries routinely. These gaps reflect a broader need for capacity-building through both domestic training and international collaboration.

Policy and training solutions

Lack of national clinical guidelines and formal policy recognition of obesity as a disease were also significant barriers, echoing findings from global analyses that demonstrate how the absence of centralized standards contributes to fragmented care delivery [14]. In countries that have successfully scaled up MBS, national policy guidelines have played a pivotal role in regulating care quality and broadening access [15]. International models such as those advocated by the International Federation for the Surgery of Obesity and Metabolic Disorders (IFSO) offer a strategic framework for training, accreditation, and outcome monitoring. These coordinated efforts are essential if Pakistan is to scale up MBS access and meet the rising burden of obesity and its complications.

Implementation-oriented solutions

Importantly, our results also indicate strong consensus on actionable solutions. Media campaigns and international fellowship programs received high agreement scores and statistically significant results indicating a shared professional belief in the value of awareness-building and skill development. Evidence supports these strategies: educational campaigns have improved bariatric referral rates in multiple settings and international collaborations have been shown to strengthen surgical programs in resource-limited countries [16,17].

Additionally, there was strong support for establishing centers of excellence and national MBS registries, both of which have been associated with improved patient outcomes, quality monitoring, and policy planning in high-performing health systems [18]. By institutionalizing bariatric care within recognized frameworks, Pakistan can ensure better surgical safety, standardized training, and long-term follow-up care. In summary, this study not only identifies statistically significant barriers to MBS in Pakistan but also offers evidence-backed, widely supported solutions. The alignment of clinical opinion with global best practices provides a strong foundation for advocacy, policy reform, and professional development.

Study limitations

This study has several limitations. The use of convenience sampling may have introduced selection bias, limiting generalizability beyond the surveyed institutions. Self-reporting bias is also possible due to the subjective nature of Likert responses. The modest sample size limited subgroup comparisons, and while statistically powered, broader insights would benefit from larger, stratified samples. Lastly, this cross-sectional design identifies perceived barriers but does not evaluate the implementation or impact of proposed solutions.

## Conclusions

Despite a growing burden of obesity and diabetes, access to MBS in Pakistan remains limited by systemic, financial, and educational barriers. The widespread professional consensus around targeted solutions such as structured training, national guidelines, and public awareness signals a strong readiness within the surgical community to advance MBS as a standard of care. Realizing this potential will require coordinated action from policymakers, educators, and healthcare leaders to integrate obesity treatment into the broader health agenda.
